# Subchronic perfluorooctanesulfonate (PFOS) exposure induces elevated mutant frequency in an *in vivo* λ transgenic medaka mutation assay

**DOI:** 10.1038/srep38466

**Published:** 2016-12-08

**Authors:** Yuanhong Chen, Wei Hu, Changjiang Huang, Shushan Hua, Qihao Wei, Chenglian Bai, Jiangfei Chen, Michelle B. Norris, Richard Winn, Dongren Yang, Qiaoxiang Dong

**Affiliations:** 1 Institute of Environmental Safety and Human Health, Wenzhou Medical University, Wenzhou 325035, P.R. China; 2 Aquatic Biotechnology and Environmental Laboratory, Warnell School of Forest Resources, University of Georgia, Athens, Georgia, USA

## Abstract

Perfluorooctanesulfonate (PFOS) has been widely detected in the environment, wildlife and humans, but few studies have ever examined its mutagenic effect *in vivo*. In the present study, we use a transgenic fish model, the λ transgenic medaka, to evaluate the potential mutagenicity of PFOS *in vivo* following a subchronic exposure of 30 days. The mutant frequency of *cII* target gene was 3.46 × 10^−5^ in liver tissue from control fish, which increased by 1.4-fold to 4.86 × 10^−5^ in fish exposed to 6.7 μg/L PFOS, 1.55-fold to 5.36 × 10^−5^ in fish exposed to 27.6 μg/L PFOS, and 2.02-fold to 6.99 × 10^−5^ in fish exposed to 87.6 μg/L PFOS. This dose-dependent increase of mutant frequency was also accompanied with mutational spectrum changes associated with PFOS exposure. In particular, PFOS-induced mutation was characterized by +1 frameshift mutations, which increased from 0% in control fish to 13.2% in fish exposed to 27.6 μg/L PFOS and 14.6% in fish exposed to 87.6 μg/L PFOS. Our findings provide the first evidence of PFOS’s mutagenicity in an aquatic model system. Given the fact that most conventional mutagenic assays were negative for PFOS, we propose that PFOS-induced mutation in liver tissue of λ transgenic medaka may be mediated through compromised liver function.

Perfluorinated chemicals (PFCs) are widely used in surfactants, lubricants, polymers, and firefighting foams^1^. Perfluorooctanesulphonicacid (PFOS) is an end product of the breakdown of many PFCs, and can enter soil, water and atmosphere. Due to its resistance to degradation in the environment and bioaccumulative characteristics, PFOS has been found globally in various living organisms including humans and wildlife^2^,^3^,^4^. Various toxicities including development toxicity, hepatotoxicity, immunotoxicity, and neurotoxicity have been reported in mammals^5^,^6^,^7^,^8^,^9^,^10^ as well as aquatic animals such as zebrafish^11^,^12^, common carp^13^,^14^, medaka^15^, and fathead minnows^16^. In particular, liver has been one of the main targets for PFOS induced toxicity^11^,^14^,^17^,^18^, and significant increase of hepatocellular adenomas has been found in Sprague-Dawley rats dietary exposed to 20 ppm PFOS for 104 weeks^19^. However, early studies with various *in vitro* genotoxic assays all showed negative findings and PFOS was thus considered as not mutagenic^19^,^20^,^21^. *In vivo* tests provide more accurate evaluation of chemical’s adverse effects on target tissues, yet whether PFOS is mutagenic *in vivo* in liver tissue has not been extensively explored.

Detection of mutations *in vivo* at endogenous loci will help advance our understanding of mutagenesis and of the role and source of mutations involved in cancer or other illness^22^. Transgenic mutation assays, unlike traditional mutation analysis *in vivo*, are sensitive, not limited to any particular tissues, and permit the screening of a large number of copies of a locus quickly^23^,^24^,^25^,^26^,^27^,^28^. The λ transgenic medaka, a transgenic fish model for *in vivo* mutation analysis, carries multiple copies of the λ bacteriophage vector that harbors the *cII* gene as a mutant target^23^. Earlier studies have shown that *cII* transgene is a reliable and sensitive mutation target that meets the fundamental requirements of mutation analysis^27^,^29^,^30^. The *cII* gene in the genome of fish has been validated to be highly responsive to mutagen exposure with mutation induction consistent with manifestation time, tissue specificity and mutational spectra^23^,^24^,^28^. In addition, the low variability in the frequencies of spontaneous mutations, highly efficient recovery of the vector and advantage of the fish to mutagen treatments, provide significant practical benefits for this model to be used in studies of *in vivo* mutation analysis^25^. Most importantly, earlier studies have demonstrated that many fundamental features of mutation analyses based on λ transgenic rodents are shared by this λ transgenic fish, providing support to the use of fish for assessing chemical risk to human health, and the uniqueness of being an aquatic model species provides additional advantages for risk assessment of aquatic pollutants. Since PFOS is an environmental toxicant that could impact wildlife including fish, this transgenic medaka is a very relevant exposure system.

In the present study, we use this λ transgenic medaka to assess the mutagenic effect of PFOS *in vivo*. Specifically, we exposed the λ transgenic medaka to various concentrations of PFOS for 30 days and the induction of *cII* mutations in the liver tissues were assessed. In addition, mutants from control and PFOS exposed fish were recovered and sequenced, and mutational spectrum was characterized. Our findings showed that PFOS exhibited weak mutagenic effect *in vivo*, and the PFOS-induced mutational spectrum was characterized by +1 frameshift mutations.

## Results

### PFOS exposure concentration

The measured concentrations of PFOS in the exposure water were 0, 6.7 ± 0.4, 27.6 ± 1.8, 87.6 ± 4.1 μg/L at day 0 and 0, 2.2 ± 0.7, 13.1 ± 1.4, 72.6 ± 3.9 μg/L at day 5 for the corresponding nominal concentrations of 0, 50, 160, 400 μg/L, respectively (Table 1). During the 30-day exposure period, low mortality was only observed at the highest exposed dose of 87.6 μg/L (2 out of 30 fish or 6.7%). No mortality or gross malformation was found in lower dose groups or control fish.

### PFOS induced *cII* mutant frequencies

**Figure 1:**
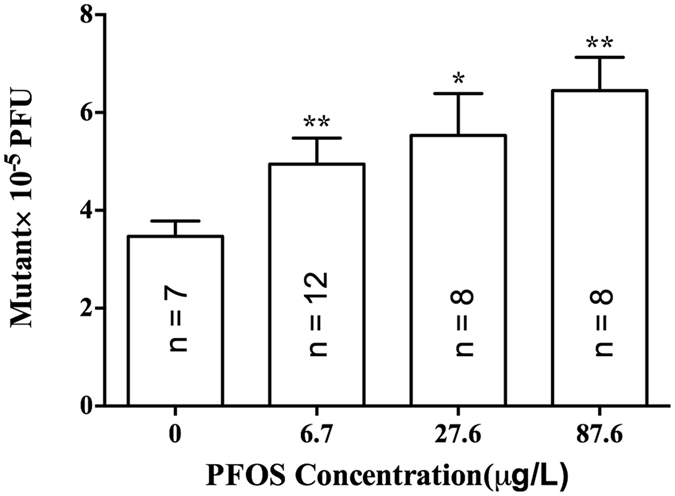
Mutant frequencies (Mean ± SE) in liver from λ transgenic medaka exposed to PFOS at 0 (0.1% DMSO), 6.7, 27.6 and 87.6 μg/L for 30 days followed by a recover period of 15 days in clean system water. *P < 0.05; **P < 0.01.

The mean mutant frequency of control fish was 3.46 × 10^−5^ (n = 7), which is similar to the spontaneous mutant frequency of (4.3 ± 0.6) × 10^−5^ observed previously in the liver tissue of λ transgenic medaka^23^. The mean mutant frequency of fish exposed to 120 mg/L ENU (positive control) was 13.7 × 10^−5^ (n = 12), which is 3.7-fold of spontaneous mutation obtained in solvent control. This value is also within the range reported previously in the liver tissue of λ transgenic medaka^23^. In PFOS exposed fish, the mean mutant frequency increased significantly when compared with control fish, and the magnitude of induction increased with elevated PFOS concentrations (Fig. 1 and Supplemental Table 1). The mutant frequencies of fish exposed to 6.7, 27.6, and 87.6 μg/L PFOS were 1.40-fold (4.86 × 10^−5^, n = 12, *P* = 0.0091), 1.55-fold (5.36 × 10^−5^, n = 8, *P* = 0.0413), and 2.02-fold (6.99 × 10^−5^, n = 8, *P* = 0.004) of the mutant frequency of control fish, respectively (Fig. 1 and Supplemental Table 1).

### PFOS induced *cII* mutational spectra

To characterize the spectra of *cII* mutations recovered from fish exposed to PFOS, a portion of the total λ c*II* was sequenced from the fish in the control, 6.7, 27.6 and 87.6 μg/L PFOS exposed groups. In the control group, forty-six plaques, representing 20.6% of the total *cII* mutants, were collected from livers of seven untreated fish (6-7 plaques per fish). Nine non-mutants were excluded, and four duplicate mutations within a single fish presumed to be of probable clonal origin, were excluded from analysis. Of the remaining thirty-three independent mutations, the spectrum of mutations was dominated by single base substitution (87.9%), of which 42.4% was transition mutation and 45.5% was transversion mutation (Table 2). Frameshift accounted for 9.1% of the mutation and all of them were -1 deletion within the homonucleotide run of guanine (nucleotides 179–184, sense stand). The remaining mutations (3%) were single base substitutions at multi-nucleotide sites.

In the 6.7 μg/l PFOS exposed group, forty-nine plaques, representing 10% of the total *cII* mutants, were collected from livers of randomly selected seven fish (7 plaques per fish). Among which, thirteen non-mutants were excluded from analysis. The spectrum of mutations in this group was significantly different from that of control (*P* < 0.05) (Table 2). We observed a lower percentage of transition mutation (22.2% vs. 42.4% in control) and a higher percentage of transversion mutation (63.9% vs. 45.5%) in this group. Frameshift mutation accounted for 5.6% of total independent mutations and all of them were +1 insertion at guanine. The remaining mutations (8.3%) were single base substitutions at multi-nucleotide sites. Further analysis focusing on individual mutation spectrum showed significantly higher percent of G/C to C/G transversion in this group (30.6%) when compared with 9.1% in the control fish (chi-squared test, *P* = 0.027).

In the 27.6 μg/l PFOS exposed group, forty-eight plagues, representing 10.3% of the total *cII* mutants, were collected from livers of seven fish (6-7 plagues per fish). Two non-mutants and seven duplicate mutants were excluded from data analysis. The spectrum of mutations in this group was similar to that of controls and high dose group, but significantly different (*P* < 0.05) from the low dose group of 6.7 μg/l PFOS (Table 2). Specifically, transversion mutation was lower (55.3% vs. 63.9% in 6.7 μg/l PFOS) and frameshift mutation was higher (18.4% vs. 5.6% in 6.7 μg/l PFOS). Further analysis focusing on +1 frameshift alone showed significantly higher percent of mutation in this group (13.2%) when compared with control fish (chi-squared test, *P* = 0.031).

In the highest treatment dose of 87.6 μg/l PFOS, forty-four plagues, representing 5.2% of the total *cII* mutants, were collected from livers of seven fish (6-7 plagues per fish). The spectrum of mutations in this group was significantly different from that of control group (*P* < 0.05), but was similar to that of 6.7 μg/l or 27.6 μg/l PFOS groups (Table 2). The most obvious change was elevated +1 frameshift mutations, accounting for 14.6% of the total independent mutations, which was significantly higher than that of control group (chi-squared test, *P* = 0.022). In addition, we also observed frameshift mutations caused by multi-base insertion and deletion (7.3%) in this treatment group. In contrast, A/T to G/C transition in this group (7.3%) was significantly lower than that of control group (chi-squared test, *P* = 0.042). Other mutations of single base substitution at multi-nucleotide sites accounted for 4.9%.

## Discussion

Our study showed elevated mutant frequencies in liver tissue of fish exposed to PFOS in a subchronic toxicity test using the λ transgenic medaka model. The mutant frequency increased with increasing PFOS concentration, suggesting chemical specific and dose-dependent mutation induction by PFOS. Analysis of spontaneous and induced mutational spectra revealed significant increase of percentage frameshift mutation in PFOS exposed fish, providing further support of PFOS-related mutation induction. To our knowledge, this is the first study reporting mutagenic effect of PFOS exposure *in* an aquatic model system, and our findings may provide new insights in understanding the carcinogenetic effects of PFOS in liver tissue.

In the present study, we found a discrepancy between nominal concentrations and the actual concentrations detected in the exposed water. This may be due to the hydrophobic nature of PFOS, which could adsorb onto tank wall when PFOS was added in water. Because PFOS concentration decreased with prolonged exposure time from day 0 to day 5, freshly made solutions were thus renewed every five days to maintain a constant PFOS exposure concentration in the fish tank. The actual PFOS concentrations tested in our study ranged from 6.7 to 87.6 μg/L, which was within the range of PFOS detected in various environmental samples (from lower than ng/L to >100 ug/L)^31^,^32^,^33^,^34^. Thus, our exposure protocol is representative and relevant for animals reside in the aquatic environment.

An earlier study with the potent mutagen benzo[a]pyrene, using the same λ transgenic medaka, reported a 14-fold induction of mutations in liver tissues with a sub-chronic exposure regime of 50 μg/L for 32 days^27^. Similarly, λ transgenic medaka exposed to pesticide 1,1-dichloropropene (DCP) at 0.44 to 16.60 mg/L for six weeks resulted in a 6- to 32-fold induction of mutations in liver tissues^24^. The highest mutant frequency observed in the present study was 2.02-fold induction in the 87.6 μg/L PFOS treatment group, suggesting that PFOS induced a relative weak mutagenic effect in λ transgenic medaka. However, our findings of PFOS mutagenic analysis met the criteria of a positive result defined for the *in vivo* transgenic mutation assay, which is “a dose response in combination with at least one point exceeding the 2× threshold”^22^ as well as the criteria specified by the Organization for Economic Co-operation and Development (OECD) in its guideline for the transgenic rodent somatic and germ cell gene mutation assays where a positive result is defined as “a dose-related increase in the mutant frequency or a clear increase in the mutant frequency in a single dose group compared to the solvent/vehicle control group^35^”. In addition, the number of fish used in the present study (7–12 fish per dose) exceeds the recommendation of 6–7 animals for detecting a 50% induction above background (power = 0.80, α = 0.05)^22^,^23^, indicating sufficient statistical power of our study design in detecting the 2× threshold mutation frequency difference.

Mutational spectrum analysis showed that PFOS treatment was associated with significant increase of G/C to C/G transversion in the low dose group, significant decrease of A/T to G/C transition in the high dose group, and significant increase of +1 frameshift mutations in the middle and high dose groups. An overall trend of dose-dependent increase was only found in the +1 frameshift mutations, suggesting a potential treatment-related effect. This finding is similar to the mutational spectrum found in the 1,1-DCP treated fish^24^. However, the majority of frameshifts were +1 insertions at thiamine and adenine in the 1,1-DCP treated fish while the majority of frameshifts in PFOS treated fish were +1 insertions at guanine in the CpG sites. In contrast, the spontaneous frameshift mutations consist primarily of −1 frameshift deletions of guanines in the string of repetitive guanines or at CpG sites^23^,^26^, which was also evidenced in the control fish of the present study. However, significant increase of −1 frameshift mutations involving the loss of G:C base pairs has also been reported in the prostate of Big Blue^®^ transgenic rats exposed to the potent mutagen of 2-amino-1-methyl-6-phenylimidazo [4,5-b] pyridine^36^. These findings revealed distinct mutational spectrum associated with different chemicals. Frameshift mutations usually lead to truncations of amino acids, which has been identified as one possible cause for inducing cancer occurrence in several cancers such as colon cancer^37^, gastrointestinal cancer^38^,^39^ and colorectal cancer^40^. It is acknowledged that comparisons between each PFOS dose level and control group across 10 individual mutation spectrum involve 30 chi-squared tests, which may result in 1-2 false positive findings based on an α value of 0.05. Thus, future studies focused on frameshift mutation alone are necessary to validate our present findings.

Although we observed a significant increase of mutant frequency and a shift of mutation spectrum in PFOS exposed fish, the exact mechanism for PFOS-induced mutagenic effects in the λ transgenic medaka is unknown. PFOS has no apparent structural alerts for mutagenicity and animal studies indicated that PFOS did not have other metabolites *in vivo*^19^. In addition, negative results were obtained in the Ames test, the reverse mutation assay using *Eschericia coli*, the *in vitro* chromosomal aberration using human whole blood lymphocytes, the *in vitro* UDS assay in rat liver primary hepatocytes, and the mouse bone-marrow micronucleus test^19^,^21^. PFOS was thus considered as not mutagenic. More recently, a new study reported that PFOS induced concentration-dependent increases in γ-H2AX foci and mutant frequencies at redBA/gam loci in transgenic mouse embryonic fibroblast cells^41^, suggesting potential genotoxicity of PFOS *in vitro*. Interestingly, the same study also reported mutation induction, though non-statistically significant, in the livers of gpt delta transgenic mice upon PFOS treatment^41^, which further supports our finding that PFOS was mutagenic *in vivo*.

Given the fact that most conventional mutagenic assays showed negative responses and all these earlier tests were performed in single cell organisms (bacteria) or mammalian cells, it is possible that PFOS itself is not mutagenic to cells *in vitro* or *in vivo*; rather PFOS may enhance mutant frequency *in vivo* through an indirect and non-genotoxic mechanism. For example, PFOS may induce mutation through its adverse effect on liver tissue function. It is known that PFOS exposure can lead to increases in liver weight, hepatic palmitoyl CoA oxidase activity, hepatic vacuolation, and hepatocellular hypertrophy in rats^19^. Our own recent study with zebrafish exposed to 0.5 μM (250 μg/L) nominal concentration of PFOS revealed severe hyperlipidemia in livers of male fish^42^. Though we did not examine the histological changes of liver tissues in medaka in the present study, we expect to see similar phenotypical changes as that of zebrafish given the similarity of these two model species (small aquaria fish) and the applied PFOS doses. We thus speculate that PFOS-induced mutant frequency in λ transgenic medaka may be mediated through adverse effects on liver tissue or more specifically the lipid oxidation in the liver^42^,^43^. The very recent study that reported PFOS’s mutagenic effect *in vitro* also observed increased lipid droplets in cells treated with PFOS and demonstrated that PFOS induced DNA double strand breaks and gene mutation was mediated by H_2_O_2_ through abnormal peroxisomal fatty acid β-oxidation^41^. Similar mechanism may underlie PFOS-induced increase of mutant frequency in the transgenic medaka model system and future studies are necessary to validate this hypothesis.

In summary, our study provides the first evidence that PFOS can be mutagenic *in vivo* and that it induced a significant increase of mutant frequency and a distinct mutational spectrum dominated by +1 frameshift mutations. Results of the present study may serve as the foundation for more extensive characterization of PFOS mutagenic effect in other tissues of the aquatic animals, and further illustrates the utility and sensitivity of the λ transgenic medaka as a model for identifying and characterizing potential genetic health risks associated with chemical exposures in the environment.

## Materials and Methods

### Animals

The λ transgenic medaka founding stocks were obtained from the Aquatic Biotechnology Laboratory, University of Georgia, Athens, GA. All fish were kept at standard laboratory condition of 28 °C on a 14:10 dark/light photoperiod in a recirculation system. Water supplied to the system was filtered by reverse osmosis (pH 7.0–7.5), and Instant Ocean^®^ salt was added to the water to raise the conductivity to 450–1000 μS/cm (system water). The fish were fed three times daily with the zebrafish diet (Zeigler, Aquatic Habitats, Apopka, FL, USA) at noon, live Artemia (Jiahong Feed Co., Tianjin, China) in the morning and evening. Animal care and uses were approved by the Institutional Animal Care and Use Committee at the Wenzhou Medical University and all methods were performed in accordance with guidelines approved by the same committee.

### Chemical stock solutions and exposure protocols

N-nitroso-n-ethylurea (ENU, CAS#759-73-9, purity >99.9%) and perfluorooctanesulphonicacid (PFOS; CAS # 1763-23-1, purity >96%), were purchased from Sigma–Aldrich Chemical (St. Louis, MO, USA). ENU, one of well-known mutagens, was used as the positive control. Stock solution of 1% ENU was prepared by dissolving 5 g ENU in 10 mmol/L sodium acetate in sterile glass vials. The ENU working solution of 120 mg/L was prepared by directly dissolving 18 mL stock solution in 1.5 L system water, and the control group was prepared by dissolving 18 mL 10 mmol/L sodium acetate in 1.5 L system water. PFOS stock solution of 20 mg/mL was prepared by dissolving it in 100% dimethyl sulphoxide (DMSO). The PFOS working solution of three concentrations (0.05, 0.16, and 0.40 mg/L) were prepared through serial dilution of the stock solution with system water. The final DMSO concentration in all treatment groups was 0.1%, and thus solvent control group received 0.1% DMSO (v/v).

For ENU exposure, fish were kept in 1.5 L working solution for 1 hr, and were then rinsed, transferred to clean system water and held for 15 days. For PFOS exposure, fish were kept in 9 L tanks in a static system for 30 days, and all tanks were covered with glass lids. Exposure water was renewed with freshly prepared solutions every 5 d. Each tank was checked for morbid fish on a daily basis, and water quality was monitored on a weekly basis. Fish were fed twice daily with freshly hatched live Artemia. Thirty fish (6 months old, ~250 mg wet weight) were randomly selected for each treatment group with 15 fish per replicate and 2 replicates per group. Generally as few as 6-7 animals per treatment group are sufficient to detect a 50% induction of mutation above background^22^,^23^. However, additional fish were included in our design to ensure we can acquire sufficient materials for analyses in the event of poor recovery of the transgene from individual liver tissue.

### Measurement of PFOS in exposure water

For quantification of PFOS in exposure tanks, 1 mL of water sample was collected from each fish tank shortly after exposure (day 0) and before renewing with fresh treatment water at day 5. Samples were stored at 4 °C, and PFOS were measured by combined liquid (Agilent 1200, American) mass spectrometry (Bruker Esquire HCT ion trap, Germany) (LC/MS) according to our previous methods^44^,^45^. Three independent replicates of each sample were prepared and analyzed. The concentrations of PFOS were calculated from standard curves and the average extraction efficiency of PFOS ranged from 75% to 80%.

### *cII* mutation assay

Mutations in the *cII* gene recovered from the livers of the λ transgenic medaka were analyzed using procedures described previously by Winn *et al*.^46^. In brief, after chemical exposure, fish were euthanized with 0.05% tricaine methanesulfonate (MS 222), and liver tissues were harvested, quickly frozen in liquid nitrogen, and stored at −80 °C. Genomic DNA was isolated from the liver tissue (~10 mg) of individual fish sequentially through digestion, extraction, and precipitation. DNA samples were re-suspended in 15–30 μL Tris-EDTA (pH 7.5) (~10–20 μg), and stored at 4 °C. The vector was recovered from fish genomic DNA (~ 10 μg) by incubation with Transpack packaging extracts (30 °C, 3 h), which simultaneously excised and packaged the vector as viable phage particles. To select *cII* mutants, the individually packaged phage were mixed with *E. coli* G1250 host strain, which carries mutant *hfl* genes that increase the stability of the cII protein facilitating a lysogenic response, and TB1 top agar and plated on 10 TB1 plates at 24 °C (60.5 °C) for 40 h. Phage with wild-type *cII* underwent lysogenization and were indistinguishable in the bacterial lawn, whereas phage with detectable mutations in the *cII* gene multiplied through the lytic cycle, forming plaques. To determine the total number of packaged phage, a subsample of the packaged phage solution was infected in G1250 cells, mixed with top agar, and incubated on three TB1 titer plates at 37 °C overnight. Mutant frequencies were calculated by dividing the total number of *cII* mutant plaque-forming units (pfu) on selective mutant screening plates by the estimated total λ positive *cII* phage on the titer plates.

### Sequence analysis of *cII* mutants

To identify mutant *cII* phenotypes, a portion of *cII* plaques was chosen from the mutant screening plates of control, 6.7, 27.6 and 87.6 μg/L PFOS treated fish. Plaques were cored randomly and purified individually on G1250 *E. coli* cells. A 410-bp λ DNA fragment, including the entire 294-bp *cII* gene, was amplified from plague lysate (1 plaque per 25 μL DNA grade water) by PCR using primer: 5′-CCACACCTATGGTGTATG-3′ and 5′-CCTCTGCCGAAGTTGAGTAT-3′ (Sunny Biotechnology Co., Ltd, Shanghai, China). PCR products were electrophoresed on 1.5% agarose gel, purified by beyotime PCR purification kit (Beyotime, Jiangsu, China), quantified by Nanodrop, and sent to Sunny Biotechnology for sequencing. *cII* mutants were verified using a Blast *cII* sequence as a reference. Multiple mutations occurring at the same nucleotide in one animal were counted as one mutation as they were considered to be representative of clonal mutant cells.

### Data analysis

The mean mutant frequencies from each treatment were compared using the COCHARM program (Troy Johnson, Proctor and Gamble, Cincinnati, OH), a modification of the generalized Cochran–Armitage test^47^. The spectra of mutations were compared among ten classes of mutations, using a program developed specifically for comparisons of mutation spectra based on the Monte Carlo method of Adams and Skopek^47^,^48^. For a specific mutation spectrum, data were sorted into target mutation type vs. rest (e.g., frameshift vs. non-frameshift) and then use Pearson’s chi-squared test to compare whether there is any significant difference between control and treatment group. A value of *P* < 0.05 was considered significant.

## Additional Information

**How to cite this article**: Chen, Y. *et al*. Subchronic perfluorooctanesulfonate (PFOS) exposure induces elevated mutant frequency in an *in vivo* λ transgenic medaka mutation assay. *Sci. Rep.* **6**, 38466; doi: 10.1038/srep38466 (2016).

**Publisher’s note:** Springer Nature remains neutral with regard to jurisdictional claims in published maps and institutional affiliations.

## Supplementary Material

Supplemental Table

## Figures and Tables

**Table 1:** Measured concentrations of PFOS in exposed water.

Nominal	Measured concentration (μg/L)	
(μg/L)	Day 0	Day 5
0	0	0
50	6.7 ± 0.4	2.2 ± 0.7
160	27.6 ± 1.8	13.1 ± 1.4
400	87.6 ± 4.1	72.6 ± 3.9

**Table 2:** Number of *cII* mutations (%) detected in liver tissues from PFOS exposed λ transgenic medaka.

Mutation Spectrum	PFOS (μg/L)			
	0^a^	6.7^b^	27.6^a,c^	87.6^b,c^
Total mutations	46	49	48	44
Independent mutations	33	36	38	41
**Transition**	**14 (42.4)**	**8 (22.2)**	**11 (28.9)**	**9 (22.0)**
G/C→A/T	6 (18.2)	4 (11.1)	8 (21.1)	6(14.6)
A/T→G/C	8 (24.2)	4 (11.1)	3(7.9)	3(7.3)
**Transversion**	**15 (45.5)**	**23 (63.9)**	**20 (55.3)**	**20 (48.8)**
G/C→T/A	5 (15.2)	10 (27.8)	12 (31.6)	8 (19.5)
G/C→C/G	3 (9.1)	11 (30.6)	3 (7.9)	4 (9.8)
T/A→A/T	1 (3.0)	0 (0.0)	3 (7.9)	5 (12.2)
A/T→C/G	6 (18.2)	2 (5.6)	2 (5.3)	3 (7.3)
**Frameshift**	**3 (9.1)**	**2 (5.6)**	**7 (18.4)**	**10 (24.4)**
+1	0 (0.0)	2 (5.6)	5 (13.2)	6 (14.6)
−1	3 (9.1)	0 (0.0)	2 (5.3)	1 (2.4)
Indel*	0 (0.0)	0 (0.0)	0 (0)	3 (7.3)
Other**	**1 (3.0)**	**3 (8.3)**	**0 (0.0)**	**2 (4.9)**

^a,b,c^Treatment groups sharing the same superscript letter are not significant different from each other based on the Monte Carlo method of Adams and Skopek.

^*^Frameshift caused by multi-base insertion and deletion.

^**^Multiplex single base substitutions at different nucleotides.

Values underlined indicate significant difference from the control group based on the Pearson’s chi-squared test (*P* < 0.05).
