# Supplementary material for: Subchronic perfluorooctanesulfonate (PFOS) exposure induces elevated mutant frequency in an in vivo λ transgenic medaka mutation assay
Source: Sci Rep. 2016 Dec 8;6:38466. doi: 10.1038/srep38466 (PMC5144067; doi:10.1038/srep38466)
Supplement: Supplemental Table [file srep38466-s1.pdf]

Subchronic perfluorooctanesulfonate (PFOS) exposure induces elevated mutant frequency in an *in vivo* λ transgenic medaka mutation assay

Yuanhong Chen[a, 1], Wei Hu[a, 1], Changjiang Huang[a,]*, Shushan Hua [a], Qihao Wei [a], Chenglian Bai[a], Jiangfei Chen[a], Michelle B. Norris[b], Richard Winn[b], Dongren Yang[a], Qiaoxiang Dong[a,] *

[a]Institute of Environmental Safety and Human Health, Wenzhou Medical University, Wenzhou 325035, PR China; [b]Aquatic Biotechnology and Environmental Laboratory, Warnell School of Forest Resources, University of Georgia, Athens, Georgia, USA

[*] Corresponding authors: cjhuang5711@163.com; dqxdong@163.con

Phone: (86) 577-86699135; Fax: (86) 577-86699135

[1] The first two authors contributed equally to this work.

Supplemental Table 1: Frequencies of cII mutants recovered from livers of control and PFOS treated λ transgenic medaka

PFOS (μg/L)	Sample ID	Total plaques screened	Mutant plaques	Mutant frequency (×10-5)	Mean mutant frequency ± S.E.M (×10-5)
0	1	1015000	29	2.86	3.46 ± 0.31
	2	1010000	40	3.96	
	3	515000	22	4.27	
	4	600000	15	2.5	
	5	785000	26	3.31	
	6	1140000	53	4.65	
	7	1380000	38	2.75	
6.7	1	395000	16	4.05	4.86 ± 0.35
	2	385000	8	2.08	
	3	1505000	67	4.45	
	4	1375000	61	4.44	
	5	865000	45	5.2	
	6	460000	15	3.26	
	7	575000	43	7.48	
	8	565000	47	8.32	
	9	535000	38	7.1	
	10	1180000	43	3.64	
	11	1415000	71	5.02	
	12	805000	35	4.35	
27.6	1	1220000	110	9.02	5.36 ± 0.96
	2	905000	69	7.62	
	3	700000	41	5.86	
	4	1995000	55	2.76	
	5	1135000	81	7.14	
	6	1120000	67	5.98	
	7	325000	11	3.38	
	8	1305000	33	2.53	
87.6	1	1315000	106	8.06	6.99 ± 0.66
	2	2340000	209	8.93	
	3	2650000	220	8.3	
	4	1265000	96	7.59	
	5	1015000	47	4.63	
	6	1390000	70	5.04	
	7	835000	39	4.67	
	8	1215000	53	4.36	
